# Application of the extended theory of planned behavior to understand Chinese students’ intention to improve their oral health behaviors: a cross-sectional study

**DOI:** 10.1186/s12889-021-12329-9

**Published:** 2021-12-19

**Authors:** Hongyan Shi, Jingya Wang, Rui Huang, Jie Zhao, Yuxin Zhang, Nan Jiang, Tetsuya Tanimoto, Akihiko Ozaki, Chunhai Shao, Jiwei Wang, Xiang He, Xiaoming Xu

**Affiliations:** 1 Dental Disease Prevention and Treatment Center of Minhang District, 1038 Fanxing Road, Huacao Town, Minhang District, Shanghai, 201100 China; 2 Minhang Branch of Fudan University School of Public health, Shanghai, China; 3 Key Lab of Health Technology Assessment of Ministry of Health, School of Public Health, Fudan University, Shanghai, China; 4 Medical Governance Research Institute, Tokyo, Japan; 5 Jyoban Hospital of Tokiwa Foundation, Fukushima, Japan; 6 Department of Clinical Nutrition, Huashan Hospital, Fudan University, Shanghai, China

**Keywords:** Theory of planned behavior, Oral health behaviors, Behavioral intention, Behavioral sciences

## Abstract

**Background:**

The present study aimed to develop and test an extended theory of planned behavior (TPB), which includes attitudes, subjective norms and perceived behavioral control, oral health knowledge, and past oral health behavior on the intention to improve oral health behaviors among primary school students in Shanghai, China.

**Methods:**

A school-based cross-sectional study was conducted with 414 students in the third-grade from 10 classes of Mingqiang Primary School located in Shanghai, China. Participants were recruited in October 2019. Data were collected through self-reported questionnaires, consisting of demographic characteristics, TPB variables, oral health knowledge and past oral health behaviors. Exploratory factor analysis was used to analyze TPB items. Pearson’s correlation and hierarchical regression analyses were conducted to identify the associated factors of intention to improve oral health behaviors.

**Results:**

The study showed that among students in the third grade, attitudes, subjective norms, perceived behavioral control, and past oral health behaviors were associated with the intention to improve oral health behaviors. In the hierarchical regression analysis, age and sex were entered in Model 1 which significantly explained 3.00% of the variance (F = 6.26, *p* < 0.01). The addition of Model 2 variables of attitudes, perceived behavioral control, subjective norms, and oral health knowledge revealed that TPB variables explained 26.70% (F = 29.59, *p* < 0.01). For Model 3, the addition of past oral health behaviors accounted for a further 1% of variance, and the full model has accounted for 28.30% of the variance with the intention to improve oral health behaviors (F = 22.8, *p* < 0.01). Regression analyses supported that among the significant variables, perceived behavioral control had the largest beta weight, followed by subjective norms and past oral health behaviors.

**Conclusion:**

The extended TPB model constructed in this study could be used to explain children’s intentions to improve oral health behaviors. Children’s oral health-related perceived behavioral control, subjective norms, and past oral health behaviors may serve as priority intervention targets in oral health promotion practices aimed at children.

**Supplementary Information:**

The online version contains supplementary material available at 10.1186/s12889-021-12329-9.

## Background

Oral health is an integral component of overall general health, self-esteem and quality of life [1]. According to the World Health Organization, “Early childhood caries continues to be a pandemic disease worldwide”, and caries is a major global public health problem. China has implemented several caries-prevention programs, such as fluoride treatments, dental sealants, oral health education and primary dental care; nonetheless, the overall prevalence of dental caries in children remains high [2]. In 2015, a national research on oral health in China showed that 38.5% of children aged 12 years had dental caries [3] .

Oral health behaviors is closely related to oral health. Adhering to oral health behaviors, for example, sensible and sound eating habits, and early and regular visits to the dentist are effective in preventing dental caries among children [4]. Poor oral health behaviors may cause the accumulation of bacterial deposits at the gingival crevice and a variety of oral health problems, which may cause an irreversible breakdown in the homeostasis of the oral ecosystem and function [5]. Therefore, a plan can be designed to promote oral health behaviors in children, thereby reducing the risk of oral health problems. A number of studies have shown that parents’ oral health behaviors, such as brushing teeth, flossing, and sugar consumption, are directly associated with their children’s corresponding behaviors [6, 7]. In addition, parents’ favorable attitudes toward controlling their children’s toothbrushing and sugar-snacking habits were also found to be associated with children’s favorable oral health behaviors [8]. Although parental influence is closely related to children’s oral health behaviors, the factors related to children themselves have received little attention, which leads to a lack of evidence for intervention in oral health behaviors from children’s perspective.

A previous meta-analysis established the theory of planned behavior (TPB) as possibly the most valid behavior change theory for predicting, describing, and understanding oral health behaviors [9]. According to the TPB, all factors that may affect behavior indirectly influence behavior through behavioral intention. Behavioral intention is determined using three variables. The first variable is attitude, which represents an individual’s overall evaluation of behavior. The second variable is subjective norms [10], which refers to a person’s beliefs about whether their significant others think they should participate in the behavior. The third variable is perceived behavioral control, which includes a person’s expectancy that behavior is within his/her control [11].

The TPB model is a flexible model, which includes opens to adding variables that can increase the explained variance. Additionally, the TPB model allows generalization to other study contexts [12]. The Integrative Behavioral Model, which combines constructs represented in the theory of reasoned action/TPB, is an emerging theory in the field of health promotion and health education [13–15]. The Integrative Behavioral Model also presents new or changed determinants that affect the intention to perform a behavior, including experiential attitude, descriptive norm, personal agency, self-efficacy, the knowledge and skills required to perform a behavior, salience of the behavior, environmental constraints, and habit [13]. Integrative Behavioral Model is still predominantly used for adults or parents of children, as they often behave in a more logical and rational way than children. To reduce the response burden on children, we added only the oral health knowledge and past oral health behaviors to the TPB constructs, and the latter was similar to the meaning of habit in the Integrative Behavioral Model. Compared with Integrative Behavioral Model, the extended TPB in this study takes behavioral intention as the dependent variable, rather than behavioral performance.

Children often rely on their parents to make decisions; therefore, the applicability of the TPB to children may be controversial. However, some studies have shown that the TPB can be used to explore the factors associated with oral health behaviors among primary school students. For example, a cross-sectional study, in which five-point Likert scale questionnaires were filled out by the students themselves, found that perceived behavioral control, subjective norms and attitudes were significantly associated with tooth-brushing intention among early primary school students (7–12 years old) [16]. A cluster randomized controlled trial in Saveh, Iran showed that the use of TPB as a framework for providing behavior-led training can be effective in promoting the oral and dental health of students in the fourth, fifth, and sixth grades in Saveh, Iran [17]. In addition, a cross-sectional study using a self-administered questionnaire with a seven-point Likert scale showed that the TPB variable was associated with moderate-to-vigorous physical activity among third to fifth grade students (aged 9 to 13) in Shanghai, China [18].

With the intensification of children’s education in China, the literacy level and comprehension ability of primary school students have also been greatly improved. Direct intervention regarding children’s oral health behaviors is feasible and necessary, rather than relying solely on parental supervision and controlling children’s behaviors. To date, most studies have explored the parental factors affecting children’s oral health behaviors; hence, there remains a gap in the exploration of children’s psychosocial or behavioral factors related to their oral health behaviors. In addition, the effects of TPB on oral health behaviors are unclear in children’s groups, and the construction of the extended TPB model is worth further exploration. Therefore, the present study aimed to develop and test an extended TPB model on children’s intentions to improve oral health behaviors, which may provide preliminary clues for future interventions to improve oral health behaviors in Chinese children.

## Methods

### Study design

This cross-sectional study was conducted in October 2019 in Minhang District, Shanghai.

### Setting and recruitment

For an exploratory factor analysis on a 27-item scale, the sample size must be at least 10 times the number of items [19]; thus, a sample size of 270 is required in this study. According to the sample size calculation method of the cross-sectional study, with the standard deviation of oral health behavior intention set at 0.62 [20], an allowable error of 0.062 and a significance level (alpha) of 0.05, the sample size required in this study was 385. therefore, choose a higher sample size requirement among the two, the final sample size of this study was not less than 385. The inclusion criteria were as follows: 1) students in the third grade; 2) no cognitive impairment; and 3) no communication disorder. A convenient cluster sampling method was adopted because of the limitations in resources and the availability of personal contacts. There are 67 primary schools in Minhang district, with approximately 826,000 students. Recruitment invitations were sent to five primary schools in Minhang district, and one primary school, Minqiang primary school was recruited on a first-come, first-served basis. Finally, with the active cooperation of the schools and reasons for cluster sampling, 10 classes were recruited out of a total of 12 classes in the third-grade. There are a total of 426 students in these 10 classes, an adequate sample size, and each student has completed a questionnaire. Informed consent was obtained from the students and their parents prior to the study. A pilot study was conducted before the main study involving 30 students in the third-grade from another primary school in Minhang district to assess readability and comprehensiveness of the questionnaire. According to the results of the pilot study, the students were able to understand most of the items and filled in the questionnaire independently. Approximately 2–3 words were beyond a few students’ comprehension which were replaced with easily understandable synonyms.

### Data collection

Questionnaires were distributed to students according to their availability during the break time (30 min). Before students filled out the questionnaire, one investigator explained the purpose of the study and the composition module of the questionnaire. Moreover, the investigator introduced the meaning of the answer options for the Likert scale to the students by drawing the corresponding emoticons on the blackboard in the classroom. As the students filled in the questionnaire, two investigators of the research team were available in the classroom to answer any issues the students might encounter while filling in the questionnaires. After completing the survey, team members were responsible for quality control by checking the questionnaires. If the number of missing values of behavioral intention (three items) was greater than one, or the number of missing values of one factor in attitude (seven items), subjective norms (five items), perceived behavioral control (five items), oral health knowledge (eight items) and past oral health behaviors (nine items) was two or above, the questionnaire can be judged invalid. Finally, 12 questionnaires were deemed as invalid, and 414 questionnaires were included in the study.

### Instruments and measures

#### Demographic characteristics

The demographic characteristics included age and sex.

#### TPB variables

The initial scale was specifically developed for this study after the research team referred to relevant literature, selected the original items, and modified them appropriately. It consists of 27 items designed to measure four dimensions: attitude (seven items), subjective norms (nine items), perceived behavioral control (seven items) and behavioral intention (four items) [see Additional file 1]. Of these, seven items of attitude and four items of subjective norms were obtained from an oral health behavior-related study [21], while the other five items of subjective norms were obtained from an oral health behavior-related, child-friendly TPB-based questionnaire [12], and seven items of perceived behavioral control, and four items of intention were obtained from a sample TPB questionnaire [22]. The translation and back-translation of the measurement scale used in this study were conducted by two proficient English speakers. The back-translated version was compared with the original English version, and differences were negotiated until the translators agreed with each other. Finally, after a discussion within the research team, the final form of the questionnaire in Chinese was established. All items were standardized on a seven-point Likert scale, ranging from 1 to 7. The mean value of each subscale was determined by dividing the total number of points on subscale items by the total number of subscale items. Therefore, the mean scores of the TPB model variables ranged from one to seven. A higher score indicated a greater level of each dimension.

#### Oral health knowledge

According to the Chinese National Oral Health Epidemiology Questionnaire, the index for oral health knowledge to reveal the status of oral health knowledge among participants consists of eight items. For example, “It is normal for the gums to bleed a little after brushing”. All items were scored with 1 = correct or 0 = incorrect, and a sum score was computed, the total score ranged from 0 to 8. A higher score indicates a higher level of knowledge about oral health.

#### Past oral health behaviors

With reference to the Chinese National Oral Health Epidemiology Questionnaire, we used nine items to measure children’s past oral health behaviors, including frequency of brushing, number of surfaces brushed, time of brushing, frequency of changing toothbrushes or replacement head, frequency of rinsing after meals, frequency of flossing, and frequency of eating sweets. Based on the existing ways of assigning weight to children’s oral health behaviors [21] and research team discussions, we initially assigned weights to the answers for each item. For example, the answer (and its weighted score) for the item “the daily frequency of brushing” is: zero (weighted score = 0), one time (weighted score = 1), and two times or more (weighted score = 3). Then, we organized two expert symposiums and invited eight experts in the field of children’s oral health to discuss the rationality of weight and put forth suggestions. Finally, the experts reached a consensus on the allocation of weights. The total score for the past oral health behaviors was added up by a weighted score for each item, which ranged from 0 to 18, with higher scores indicating higher levels of children’s oral health behaviors.

### Questionnaire modifications

According to Shanghai’s Chinese curriculum standards for primary schools, students in the third-grade are required to learn about 2000 common words and be able to correctly write more than 1000 words. To ensure the readability of the questionnaire for students in the third-grade, we made appropriate modifications to the items of the questionnaire by referring to the vocabulary required by the Chinese textbook for students in the third-grade in Shanghai (readers can view the questionnaire in Supplementary Appendix 1).

#### Ethical considerations

Ethics approval of this study was granted by the ethics committee of the Minhang Branch of Fudan University Stomatology Disease Center in Shanghai, China.

#### Data analysis

EpiData Entry was used for data entry and documentation. The Statistical Package for the Social Sciences (SPSS version 21.0) was used for data management and analysis. Descriptive statistics and frequency distributions were calculated to describe the sociodemographic profiles of the study participants. Construct validity was examined using exploratory factor analysis. A principal component analysis with a varimax rotation was applied to extract the factors. A hierarchical multiple regression was used to explore the relationship of independent variables with the intention to oral health behaviors. Based on strong theoretical and logical framework, hierarchical multiple regression allows the researcher to accumulate input independent variables. Hierarchical multiple regression analysis for the intention to improve oral health behaviors was performed by entering independent variables into 3 separate models cumulatively. Model 1 consisted of sex and age, which were defined as the demographic characteristics and was entered first. Model 2 was comprised of scores from the attitudes, subjective norms, and perceived behavioral control; these were TPB variables. Model 3 consisted of scores from the oral health knowledge and past oral health behaviors; these were extra extended TPB variables. This sequential entry order of variables was based on two priori hypotheses, first, the additional variance may be explained by extended TPB variables after accounting for the variance related to individual factors. Second, the additional variance may be explained by oral health knowledge and past oral health behaviors after accounting for the variance related to individual factors and TPB variables. The model fit of a single regression model was determined by the value of the coefficient R^2^ of the model (*P* < 0.05). The model fit of the hierarchical multiple regression was judged by the change in R^2^ (*P* < 0.05). All tests were two-tailed.

## Results

### Demographic characteristics of participants

The participants of this study were 414 students in the third-grade in primary school; 210(50.72%) were male, and 204(49.28%) were female. The mean age was 10.38 (SD = 0.48) years.

### Exploratory factor analysis and descriptive analysis of the TPB variables

An exploratory factor analysis was performed by introducing all the TPB variables, excluding oral health knowledge and past oral health behaviors. The Kaiser-Meyer-Olkin measure (KMO value of 0.90) and Bartlett’s test (χ2 = 5233.92, *p* < 0.001) confirmed the adequacy of the exploratory factor analysis. Four factors with eigenvalues > 1 were retained, which explained 69.80% of the total variance. The factor loadings and Cronbach’s alpha for these scales and items are presented in Table 1. Table 2 lists four factors with eigenvalues > 1 and their corresponding variance contribution rate and cumulative variance contribution rate.

**Table 1:** Item content, Factor loading and reliability coefficient of the TPB variables

item	Item loading	Reliability coefficient
Attitude		0.917
Protecting oral health makes me feel unbeneficial/beneficial	0.811	
Protecting oral health makes me feel stupid/clever	0.836	
Protecting oral health makes me feel unpleasant/pleasant	0.794	
Protecting oral health makes me feel unenjoyable/enjoyable	0.740	
Protecting oral health makes me feel useless/useful	0.862	
Protecting oral health makes me feel unimportant/important	0.830	
Protecting oral health makes me feel unadvisable/ advisable	0.743	
Subjective norms		0.873
My teacher thinks that I should adopt oral self-care behaviors	0.817	
My classmates think that I should adopt oral self-care behaviors	0.838	
My teacher’s opinion of oral health is important	0.826	
My classmates’ opinion of oral health is important	0.808	
I care what my teachers think I should do in oral health	0.627	
Perceived behavioral control		0.883
I find it difficult to take care of my teeth due to the lack of school health education	0.859	
I find it difficult to take care of my teeth due to lack of reminders	0.836	
I find it difficult to take care of my teeth due to the lack of oral hygiene awareness	0.856	
I am still confident to complete daily oral health behaviors even if there is no reminder from others	0.661	
I am still confident to complete daily oral health behaviors even I am lack of oral hygiene awareness	0.627	
Behavioral intention		0.829
I intend to brush my teeth twice a day in the next 12 months	0.818	
I intend to gargle after eating in the next 12 months	0.843	
I intend to have regular oral examinations in the next 12 months	0.786

**Table 2:** Eigenvalues, corresponding variance contribution rate and the cumulative variance contribution rate of the questionnaire

Construct	Range (low-high)	eigenvalues ​	Percentage variance explained(%)	cumulative Percentage variance explained(%)
Attitudes	1–7	7.434	37.168	37.168
Subjective norms	1–7	2.868	14.340	51.508
Perceived behavioral control	1–7	2.129	10.644	62.152
Behavioral intention	1–7	1.528	7.638	69.790

For details of which items were included in each construct, see Table 1

Factor 1 contains the most information and can explain 37.17% of the total variation. Seven items measured attitudes toward oral health behaviors, which assessed the expected value of engaging in daily oral health behaviors; it was named as “Attitude”. Factor 2 contains five items, which can explain 14.34% of the total variation, and was named as “subjective norms”. Factor 3 contains five items, which measured perceived behavioral control using the mean value of these items and can explain 10.64% of the total variation, it was named “perceived behavioral control”. Factor 4 contains three items, which can explain 7.64% of the total variation, and is named “Behavioral intention”. In addition, all factor loadings were above 0.600, which is efficient. This accounted for 69.79% of the total variance. Item analysis for attitudes, subjective norms, perceived behavioral control, and behavioral intention showed high internal reliability Cronbach’s alpha coefficients of 0.917, 0.873, 0.883, and 0.829, respectively, and the total reliability of the questionnaire was 0.907, which passed the reliability test. Table 3 describes the mean scores of the participants’ extended TPB components.

**Table 3:** Mean ± sd and intercorrelations among the extended TPB variables

Study variables	Mean ± sd	Correlation coefficients†					
		Behavioral intention	Attitudes	Subjective norms	Perceived behavioral control	Oral health knowledge	Past oral health behavior
Behavioral intention	6.13 ± 1.32	–	–	–	–	–	–
Attitudes	6.01 ± 1.36	0.277^**^					
Subjective norms	5.40 ± 1.58	0.408^**^	0.321^**^				
Perceived behavioral control	5.72 ± 1.46	0.418^**^	0.403^**^	0.373^**^			
Oral health knowledge	4.13 ± 2.41	0.072	0.064	0.083	0.040		
Past oral health behavior	9.90 ± 2.52	0.237^**^	0.179^**^	0.221^**^	0.127^**^	0.171^**^	

^**^P < 0.01, ^*^P < 0.05, †Pearson’s correlation

### Correlations of the extended TPB variables

The correlation and mean of the extended TPB variables are shown in Table 3. Behavioral intention was significantly and positively correlated with attitude, subjective norms, perceived behavioral control, and past oral health behaviors. The other components of the model also showed significant correlations with each other.

### Hierarchical regression analysis accounts for variance of extended TPB

Hierarchical multiple regression analyses were conducted to identify whether TPB, past oral health behaviors, and oral health knowledge were associated with the intention to improve oral health behaviors (Table 4). In Model 1, the variables of age and sex significantly accounted for 3.00% of the variance (F = 6.26, *p* < 0.01). Variables of attitudes, perceived behavioral control and subjective norms were entered into Model 2, which increased the proportion of variance to 26.70% (F = 29.59, *p* < 0.01). In Model 3, perceived behavioral control, subjective norms and past oral health behaviors were found to be associated with the intention to improve oral health behaviors. The standardized regression coefficient of perceived behavioral control was the largest (B = 0.26), followed by subjective norms (B = 0.21) and past oral health behaviors (B = 0.06). There was no evident difference between perceived behavioral control and subjective norms in relation to the intention to improve oral health behaviors. The full model accounted for 28.30% of the variance (F = 22.8, *p* < 0.01). The collinearity statistics revealed that the variance inflation factor ranged from 1.03–1.30.

**Table 4:** Hierarchical multiple regression analysis including age, sex, attitudes, subjective norms, perceived behavioral control, oral health knowledge and past oral health behavior

Variables	Model1		Model2		Model3	
	B (SE)	β	B (SE)	β	B (SE)	Β
intercept	4.16(1.40)^**^		3.53(1.23)^**^		3.03(1.23)^**^	
Sex	0.43(0.13)^**^	0.16^*^	0.22(0.11)	0.08	0.21(0.13)	0.08
Age	0.13(0.14)	0.05	−0.08(0.12)	−0.03	−0.08(0.12)	−0.03
Attitudes			0.07(0.05)	0.07	0.05(0.05)	0.05
Subjective norms			0.23(0.04)^**^	0.28^**^	0.21(0.04)^**^	0.25^**^
Perceived behavioral control			0.27(0.04)^**^	0.29^**^	0.26(0.04)^**^	0.28^**^
Oral health knowledge					0.02(0.03)	0.03
Past oral health behavior					0.06(0.02)^**^	0.12^**^
R^2^	0.030^**^		0.267^**^		0.283^**^	
F	6.262^**^		29.59^**^		22.80^**^	
ΔR^2^	0.030^**^		0.237^**^		0.016^*^	
ΔF	6.262^**^		43.84^**^		4.536^*^	

^**^P < 0.01, ^*^P < 0.05; *B* standardized regression coefficient; *SE* standard error of standardized regression coefficient; *β* unstandardized regression coefficient

## Discussion

The present study explored the associated factors of intention to improve oral health behaviors in Chinese students based on extended TPB. The correlation analysis findings demonstrate that intention to improve oral health behaviors was positively correlated with attitudes, subjective norms, perceived behavioral control, and past oral health behaviors, which is in line with other studies in this field [23]. The final hierarchical model showed that subjective norms, perceived behavioral control, and past oral health behavior were associated with intention to improve oral health behaviors. This is consistent with the findings from previous studies, which indicated that TPB variables accounted for less than 34% of the variance in oral health behaviors [23].

In this study, although attitude was positively correlated with intentions, it had no association with the intention to engage in oral health behaviors in our hierarchical regression model. This may be because of a causal lag [24]. If there is a relatively long causal lag, the change in behavior intention produced by a change in attitudes does not occur until after an interval period. In the future, intervention studies are needed to verify the relationship between attitude and behavioral intention.

The perceived behavioral control component demonstrated the strongest effect on intention to improve oral health behaviors in this study, which is consistent with the findings of a meta-analysis that perceived behavioral control is the strongest predictor of health behavior in the TPB model [21]. In addition, a TPB-based intervention study on younger children aged 7–12 years showed that perceived behavioral control was the most favorable variable for brushing behaviors in children [16]. It is undeniable that for children in the third grade, parental will and behaviors have an important impact on children’s oral health behaviors. For example, many parents would force children with no intention to improve oral health behaviors to perform basic oral care. Conversely, some children may intend to engage in some oral health behaviors whereas their parents may not provide for their children’s needs. However, children’s oral health behaviors involve many aspects such as brushing teeth and eating snacks, which makes it difficult for parents to continuously supervise their children. Therefore, a high level of perceived behavioral control is also important for children’s self-management of oral health behaviors. Further experimental studies should be conducted in the future to examine whether oral health behaviors can be improved by improving children’s perceived behavioral control levels.

Subjective norms was considered to be the weakest predictor of intention [25]; however, our results show that subjective norms is not only consistently positively correlated with intention, but also explain intention to a good extent. The positive relationship between subjective norms and intention to improve oral health behaviors may be explained by the teachers’ and classmates’ views on oral health behaviors. Practically, this study revealed that subjective norms is important in the extended TPB model, which demonstrates possible directions for the oral health behaviors of students. Owing to the obvious impact of subjective norms, it can be assumed that directing social change in a positive direction will be effective in emphasizing the environmentally-oriented behavior of individuals [26]. This can refer to people who have an impact on a large crowd (e.g., teachers, and classmates). Similarly, emphasizing the importance and benefits of oral health behaviors can increase the number of students who protect their teeth, because students will be more inclined to engage in behaviors that a growing number of the crowd engages in. Therefore, appropriate training of teachers and parents regarding oral health education is critical. In addition, the supervision of harmful oral habits among students provided by teachers, classmates and family members will be effective.

Some studies have revealed that oral health knowledge is positively associated with good practice of oral health maintenance and behavioral intentions among people over 12 years old [21, 27–29]. However, our study showed that oral health knowledge was not associated with the intention to improve oral health behaviors of children in the third-grade. Similar to our study, one study showed that oral health knowledge was not associated with brushing frequency in low-income African American children in grades 5 to 8 [30]. Therefore, the relationship between oral health knowledge and oral health-related behavioral intention remains controversial.

The results of this study revealed a positive relationship between past oral health behaviors and the intention to improve oral health behaviors. Previous research has shown the effect of past behavior on decision-making intentions among consumers within the TPB [31]. Past behavior guides future responses through two processes [32]; when an action is frequently performed, it can be performed relatively automatically, so that past behavior is positively correlated to behavior intention. In addition, the frequency of past behavior reflects habit strength and has a direct effect on behavioral intention. A meta-analysis [33] showed that the frequency of past behavior could indirectly affect intention through its effects on attitude, subjective norms, and perceived behavioral control, and past behavior also has direct effects on intentions independent of these other variables.

Studies have shown that Integrative Behavioral Model using additional constructs, accounts for more variance than TPB alone [34, 35]. This is consistent with the results of the present study. However, compared with Integrative Behavioral Model, only two variables, oral health knowledge and past oral health behaviors, were included in the extended TPB model of this study. It is worth noting that other constructs within Integrative Behavioral Model such as self-efficacy and environmental factors (parental influences and oral health education in schools) may have a significant impact on children’s health behaviors. It is necessary to further explore the possible factors influencing the oral health behaviors of young children based on Integrative Behavioral Model.

The present study has some limitations that need to be addressed in further studies. First, the sample is a non-probability sample of students in the third-grade from only one school. Therefore, the results of this study have limited generalizability to students in Minhang district or other districts in Shanghai. Second, the use of self-report questionnaires may have led to measurement bias. In addition, this study did not investigate the education levels of students’ guardians and socioeconomic status, as it was difficult to derive because the students filled in the information. Third, this is a cross-sectional study, hence, the causal relationship between the factors and behavioral intention needs to be further explored. Fourth, we used a seven-point Likert scale to measure variables related to children’s oral health behaviors. Likert responses may raise problems among children [36], we thus introduced the meaning of the options by drawing the corresponding emoticons before the students filled out the questionnaires. Less than 10% of the students expressed doubts when completing the questionnaire, and they were able to successfully complete the questionnaire with the help of the surveyor. Moreover, each of the TPB constructs proved to have good reliability, reflecting that students can accurately understand the meaning of the item. In future surveys involving young children, it would be more efficient to directly add illustrative pictures to the answers for each item in the questionnaire. Finally, although the expanded TPB model could be used to explain the intention to improve oral health behaviors, the appropriate components of other behavioral change theoretical models such as Integrative Behavioral Model and the health belief model have not been used in combination in this study. Future studies could include other variables associated with children’s oral health behaviors, such as parental oral health behaviors [6, 7] and parental attitudes toward their children’s oral health behaviors [8].

## Conclusion

The extended TPB model constructed in this study could be well used to explain the children’s intention to improve oral health behaviors. Children’s oral health related perceived behavioral control, subjective norms and past oral health behaviors may serve as the priority intervention targets in oral health promotion practices aiming at children’s groups.

## Supplementary Information

**Additional file 1.** Child Caries Prevention Questionnaire, this document contains the questionnaire we used in this study.

## Data Availability

The datasets generated and analyzed during the past study are not publicly available because of privacy or ethical restrictions but are available from the corresponding author upon reasonable request.

## Abbreviations

TPB: Theory of planned behavior
