# Supplementary material for: Application of the extended theory of planned behavior to understand Chinese students’ intention to improve their oral health behaviors: a cross-sectional study
Source: BMC Public Health. 2021 Dec 19;21:2303. doi: 10.1186/s12889-021-12329-9 (PMC8684633; doi:10.1186/s12889-021-12329-9)
Supplement: Supplementary file 1 — Additional file 1. Child Caries Prevention Questionnaire, this document contains the questionnaire we used in this study. [file 12889_2021_12329_MOESM1_ESM.docx]

**Child Caries Prevention Questionnaire**

Hi guys! To further improve preventive oral health care for children and adolescents, we are interested in your thoughts and practices about preventive oral health care. This survey is not related to your academic performance, and the results of the survey will not be shared with parents or teachers. We hope that you will answer truthfully according to the questions. Thank you!

Requirement: please tick the appropriate option for each question.

**Part One**

**I. General information (single-choice questions unless otherwise specified)**

1. Your sex

1)male 2) female

2. You are age in years

**II. Oral status (single-choice questions unless otherwise specified)**

1. What is your brushing habits ? (If item 4 is selected, fill in the lines)

1) Brush twice a day or more

2) Brush once a day in the morning

3) Brush 1 time per night

4) Other

2.What parts of your teeth do you usually brush? (Multiple answers available for this question)

| 1) Outer side of teeth | 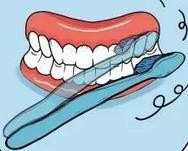 |
| --- | --- |
| 2) Medial side of posterior teeth | 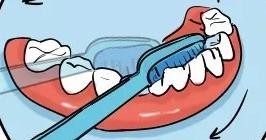 |
| 3) Medial side of front teeth | 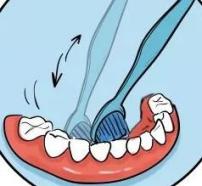 |
| 4) Occlusal surface | 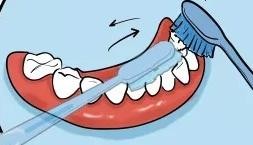 |
| 5)unknown |  |

3. How long do you usually spend brushing your teeth?

1) <1 minute 2) ≥1 minute and <2 minutes 3) ≥2 minutes and <3 minutes

4) ≥3 minutes and <4 minutes 5) ≥4 minutes

4.How often do you usually replace your toothbrush or replacement head?

1) One month or less 2) One month to three months inclusive

3) Three months to six months inclusive 4) More than six months

5. Do you rinse your mouth after meals?

1) Never rinsed mouth after meals 2) Sometimes rinsed mouth after meals (<50% frequency)

3) Always rinsed your mouth after meals (frequency ≥ 50%) 4) Rinsed your mouth after all meals

5) Rinsed your mouth after every meal (including snacks)

6. Do you floss your teeth after meals?

1) Never flossed after meals 2) Sometimes flossed your teeth after meals (<50% frequency)

3) Always flossed your teeth after meals (frequency ≥ 50%) 4) Flossed your teeth after all meals

5) Flossed your mouth after every meal (including snacks)

7.How much of the following food or drink do you normally consume? (please tick the appropriate option)

|  | never | 1 to 3 times a month | 1 time per week | 2-6 times per week | 1 time per day | ≥2 times per day |
| --- | --- | --- | --- | --- | --- | --- |
| Frequency of eating desserts and candies (biscuits, cakes, etc.) |  |  |  |  |  |  |
| Frequency of drinking sweets (sugar water, cola and other carbonated beverages, orange juice, apple juice, lemonade, etc.) |  |  |  |  |  |  |
| Frequency of eating sweetened milk, milk powder, tea, soy milk |  |  |  |  |  |  |

**Part Two**

| 1. **Attitude**   **Please read each statement carefully and select the option between 1 and 7 that matches your feelings and put a tick on the line.** |
| --- |
| Protecting oral health makes me feel |
| 1.Unbeneficial : 1 : 2 : 3 : 4(Neutral) : 5 : 6 : 7 : beneficial |
| 2.Stupid : 1 : 2 : 3 : 4(Neutral) : 5 : 6 : 7 : clever |
| 3.Unpleasant: 1 : 2 : 3 : 4(Neutral) : 5 : 6 : 7 : pleasant |
| 4.Unenjoyable: 1 : 2 : 3 : 4(Neutral) : 5 : 6 : 7 : enjoyable |
| 5.Useless : 1 : 2 : 3 : 4(Neutral) : 5 : 6 : 7 : useful |
| 6.Unimportant: 1 : 2 : 3 : 4(Neutral) : 5 : 6 : 7 : important |
| 7.Unadvisable: 1 : 2 : 3 : 4(Neutral) : 5 : 6 : 7 : advisable |
| 1. **Subjective norms**   **Please read each statement carefully and select the option between 1 and 7 that matches your feelings and put a tick on the line.** |
| 1.My parents thinks that I should adopt oral self-care behaviors |
| Very inconsistent : 1 : 2 : 3 : 4(Neutral) : 5 : 6 : 7 : very consistent |
| 2.My teacher thinks that I should adopt oral self-care behaviors |
| Very inconsistent : 1 : 2 : 3 : 4(Neutral) : 5 : 6 : 7 : very consistent |
| 3.My classmates think that I should adopt oral self-care behaviors |
| Very inconsistent : 1 : 2 : 3 : 4(Neutral) : 5 : 6 : 7 : very consistent |
| 4. My parents’ opinion of oral health is important |
| Very inconsistent : 1 : 2 : 3 : 4(Neutral) : 5 : 6 : 7 : very consistent |
| 5. My teacher’s opinion of oral health is important |
| Very inconsistent : 1 : 2 : 3 : 4(Neutral) : 5 : 6 : 7 : very consistent |
| 6. My classmates’ opinion of oral health is important |
| Very inconsistent : 1 : 2 : 3 : 4(Neutral) : 5 : 6 : 7 : very consistent |
| 7. I care what my parents think I should do in oral health |
| Very inconsistent : 1 : 2 : 3 : 4(Neutral) : 5 : 6 : 7 : very consistent |
| 8. I care what my teachers think I should do in oral health |
| Very inconsistent : 1 : 2 : 3 : 4(Neutral) : 5 : 6 : 7 : very consistent |
| 9. I care what my classmates think I should do in oral health |
| Very inconsistent : 1 : 2 : 3 : 4(Neutral) : 5 : 6 : 7 : very consistent |
| 1. **Perceived behavioral control**   **Please read each statement carefully and select the option between 1 and 7 that matches your feelings and put a tick on the line.** |
| 1. I find it difficult to take care of my teeth due to the lack of school health education |
| Very inconsistent : 1 : 2 : 3 : 4(Neutral) : 5 : 6 : 7 : very consistent |
| 2. I find it difficult to take care of my teeth due to lack of reminders |
| Very inconsistent : 1 : 2 : 3 : 4(Neutral) : 5 : 6 : 7 : very consistent |
| 3. I find it difficult to take care of my teeth due to lack of oral hygiene awareness |
| Very inconsistent : 1 : 2 : 3 : 4(Neutral) : 5 : 6 : 7 : very consistent |
| 4. I am still confident to complete daily oral health behaviors even if there is school health education |
| Very inconsistent : 1 : 2 : 3 : 4(Neutral) : 5 : 6 : 7 : very consistent |
| 5. I am still confident to complete daily oral health behaviors even if there is no reminder from others |
| Very inconsistent : 1 : 2 : 3 : 4(Neutral) : 5 : 6 : 7 : very consistent |
| 6. I am still confident to complete daily oral health behaviors even I am lack of oral hygiene awareness |
| Very inconsistent : 1 : 2 : 3 : 4(Neutral) : 5 : 6 : 7 : very consistent |
| 7. I can participate in school oral health promotion activities if I want |
| Very inconsistent : 1 : 2 : 3 : 4(Neutral) : 5 : 6 : 7 : very consistent |
| 1. **Behavioral intention**   **Please read each statement carefully and select the option between 1 and 7 that matches your feelings and put a tick on the line.** |
| 1.I intend to brush my teeth twice a day in the next 12 months |
| Very inconsistent : 1 : 2 : 3 : 4(Neutral) : 5 : 6 : 7 : very consistent |
| 2.I intend to gargle after eating in the next 12 months |
| Very inconsistent : 1 : 2 : 3 : 4(Neutral) : 5 : 6 : 7 : very consistent |
| 3.I intend to have regular oral examinations in the next 12 months |
| Very inconsistent : 1 : 2 : 3 : 4(Neutral) : 5 : 6 : 7 : very consistent |
| 4.I intend to participate in school oral health education activities in the next 12 months |
| Very inconsistent : 1 : 2 : 3 : 4(Neutral) : 5 : 6 : 7 : very consistent |
| 1. **Oral health knowledge**   **Please read each statement carefully and tick “□” you think is correct.** |
| 1. It's normal for your gums to bleed when you brush your teeth |
| □True □False □I don’t know |
| 2. Bacteria can cause inflammation of the gums |
| □True □False □I don’t know |
| 3. Brushing does not help prevent gum inflammation |
| □True □False □I don’t know |
| 4. Bacteria can cause dental caries |
| □True □False □I don’t know |
| 5. Eating sugar can lead to tooth decay |
| □True □False □I don’t know |
| 6. Fluoride is useless for protecting teeth |
| □True □False □I don’t know |
| 7. Fissure sealing can protect teeth |
| □True □False □I don’t know |
| 8. Oral diseases may affect general health |
| □True □False □I don’t know |
